# Natural history of Impella 5.5 in patients with cardiogenic shock and small left ventricular cavities

**DOI:** 10.3389/fcvm.2026.1893068

**Published:** 2026-08-05

**Authors:** Aarti Desai, Hans Mautong, Peter Wlodkowski, Rebecca Klingbeil, Cetin Alak, Jose Ruiz, Anna Shapiro, Archer Martin, Jose Nativi, Rohan Goswami

**Affiliations:** 1 Division of Heart Failure and Transplantation, Mayo Clinic, Jacksonville, FL, United States; 2 Internal Medicine Resident, John H. Stroger Jr. Hospital of Cook County, Chicago, IL, United States; 3 School of Health, Universidad Espíritu Santo-Ecuador, Samborondón, Guayas, Ecuador; 4 Department of Anesthesia and Peri-operative Medicine, Mayo Clinic, Jacksonville, FL, United States; 5 Department of Cardiology, Uludag University School of Medicine, Bursa, Türkiye

**Keywords:** cardiogenic shock, efficacy, heart failure, Impella 5.5, safety, small left ventricle

## Abstract

The Impella 5.5 is an axillary mechanical circulatory support device used in patients with cardiogenic shock, but those with small left ventricular (LV) cavities may be at risk for suction events, hemolysis, malpositioning, hypoperfusion, and LV injury. Informally accepted guidelines, extrapolated from durable LVAD studies, suggest a minimum left ventricular internal dimension in diastole (LVIDd) of 58 mm, with some clinical practices using a more stringent ≤52 mm cutoff. We retrospectively analyzed 154 consecutive patients supported with Impella 5.5 between January 2020 and December 2024. Overall, 38 patients had an LVIDd ≤58 mm, and 116 had an LVIDd >58 mm; a key subgroup of 21 patients presented with a very small LVIDd of ≤52 mm]. Baseline demographics, comorbidities, body mass index, and renal function were similar [across groups], although those with larger LVIDd had lower ejection fractions (16% vs. 24%, *p* = 0.001). Median support duration was 24 days in all groups, and pre- and post-implant hemodynamic and laboratory improvements were comparable. Rates of transplantation (79% vs. 90%, *p* = 0.088) and LVAD implantation (2% vs. 6%, *p* = 0.159) did not differ significantly in the ≤58 mm cohort. Notably, within the ≤52 mm subgroup, 90-day survival remained high at 90.5% (*p* = 0.336), with significant improvements in right atrial pressure (17 to 10 mmHg, *p* = 0.041), mean pulmonary artery pressure (36 to 26 mmHg, *p* = 0.008), and eGFR (45 to 57 mL/min/1.73m^2^, *p* = 0.003). Remarkably, native cardiac recovery was observed exclusively in the smaller LV cohorts, occurring in 11% of the ≤58 mm group (*p* = 0.003 vs. 0%, *p* = 0.003) and reaching 19% in the ≤52 mm group (vs. 0% in larger cavities, *p* < 0.001), while durable LVAD escalation was entirely avoided in the ≤52 mm cohort (0% vs. 6%). Ninety-day event-free survival was excellent across all cohorts 92% vs. 96% in the primary analysis, with no major device-related complications, suction events, clinically significant hemolysis, bleeding, stroke, or escalation to Bi-VAD support. These findings strongly support the safety and feasibility of Impella 5.5 in extremely constrained, small LV cavities.

## Graphical Abstract

**Figure:**
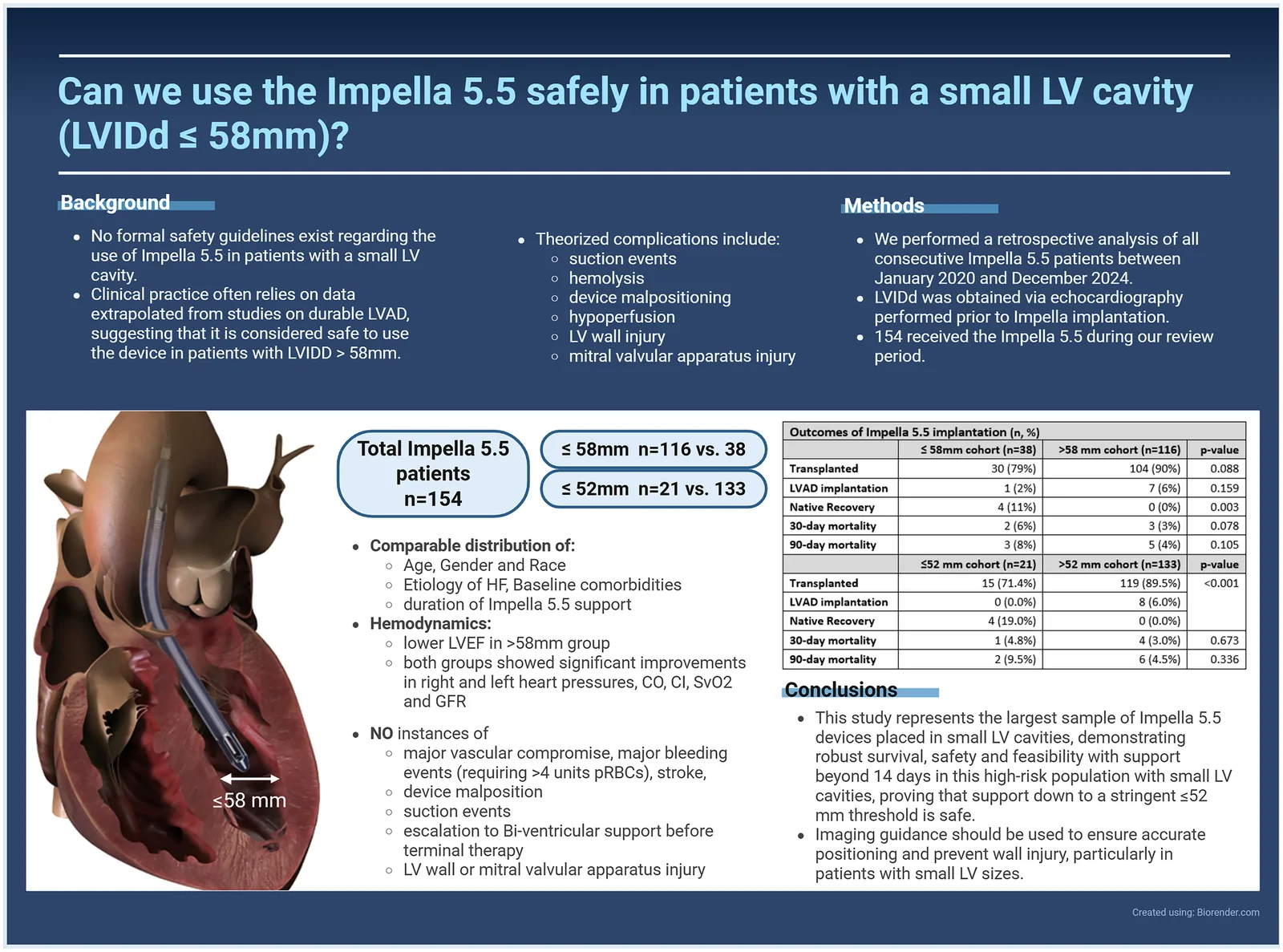

## 1 Background

Heart failure (HF) remains one of the leading causes of morbidity and mortality, with advanced stages often requiring mechanical circulatory support (MCS) to maintain hemodynamic stability and end-organ perfusion. The Impella 5.5 (Abiomed, Danvers, MA, USA) is a trans-valvular, axillary micro-axial mechanical circulatory support (aMCS) device designed to provide hemodynamic support in patients with cardiogenic shock (CS). The Impella 5.5 pumps blood from the left ventricle (LV) into the aorta continuously with flow rates up to 6 L/min, thereby unloading the LV and improving end-organ perfusion while decreasing both the LV wall stress and myocardial oxygen demand. This intervention serves as a bridge to durable left ventricular assist device (LVAD) implantation, heart transplantation and, in some cases, native organ recovery (1).

Despite its growing utilization, data on the safety and efficacy of the Impella 5.5 in specific subsets of patients, such as those with a small LV cavity, remain limited. In 2,022, a study consisting of 313 durable LVAD patients, reported worse survival if the left ventricular end-diastolic diameter (LVEDD) was <59 mm. Patients with a smaller LVEDD had lower survival compared to larger LVEDD patients (71% vs. 85% at 1 year and 58% vs. 80% at 2 years, *p* = 0.003) (2). Extrapolated from this study, the informally recognized best practices suggest a minimum left ventricular internal dimension in diastole (LVIDd) of >58 mm for the use of Impella 5.5. It is theorized that in patients with small LV cavities, complications such as suction events, hemolysis, malpositioning, hypoperfusion, LV wall injury, and mitral valvular apparatus injury may occur (2, 3). However, the impact of left ventricular size with the use of Impella 5.5 remains unexplored. We aim to study the safety, efficacy and outcomes of Impella 5.5 in HF patients with small LV cavities, defined by left ventricular internal dimension in diastole (LVIDd, presenting with cardiogenic shock.

## 2 Methods

### 2.1 Study design and population

We performed a retrospective analysis of all consecutive patients who received Impella 5.5 implantation at Mayo Clinic in Florida between January 2020 and December 2024. LVIDd was obtained via echocardiography performed prior to Impella implantation. 154 received the Impella 5.5 during our review period.

To thoroughly evaluate the impact of left ventricular size on clinical and hemodynamic outcomes, patients were stratified using two distinct clinical thresholds. For the primary cohort analysis, we utilized the informally accepted clinical cutoff derived from durable LVAD studies: 38 patients had a small LV cavity defined as an LVIDd of ≤58 mm, and 116 had an LVIDd of >58 mm. For the secondary, more stringent subgroup analysis, we stratified the cohort using a tighter, informally recognized clinical threshold of ≤52 mm, yielding 21 patients with an extremely small LV cavity (LVIDd ≤52 mm) and 133 patients with an LVIDd >52 mm.

### 2.2 Data collection

Patient demographics, perioperative data, hemodynamic parameters, Impella support timeline, and outcomes were collected using electronic medical records. The standard of care at our institution for patients implanted with the Impella 5.5 includes the placement of a peripherally inserted central catheter (PICC) to monitor central venous pressure and other hemodynamic parameters, as well as to record mixed venous saturation. Baseline laboratory values were collected at the time of admission, and baseline hemodynamics were collected via a right heart catheterization post-admission. Post-Impella hemodynamics and laboratory values were collected 72 h post-Impella implantation.

### 2.3 Statistical analysis

All statistical analyses were performed using the statistical package SPSS (version 23.0; SPSS Inc., Chicago, Illinois). Descriptive analysis of categorical variables was presented as frequencies and percentages. Quantitative non-normally distributed data was presented as a median with an interquartile range.

The same statistical protocol was applied independently to cohort comparisons: both ≤58 mm vs. > 58 mm and ≤52 mm vs. > 52 mm. The Mann–Whitney U test was used to compare quantitative data, and the chi-square test was used to compare longitudinal data and pre- and post-Impella hemodynamics and laboratory values between the two groups. Statistical significance was defined by a *p*-value of < 0.05.

### 2.4 Ethical considerations

This study was conducted in accordance with the Declaration of Helsinki and was approved by the Mayo Clinic Institutional Review Board. Personal data protection was ensured by maintaining patient confidentiality and anonymity.

## 3 Results

During the review period, 154 patients were supported with Impella 5.5 and are included in the study. Of these, 38 patients had an LVIDd ≤ 58 mm and 116 patients have LVIDd >58 mm.

### 3.1 Baseline characteristics

The median age was similar between groups, 60 (53–66) years in the ≤58 mm group vs. 57 (49–65) years in the >58 mm group, *p* = 0.196 (Table 1). Our study cohort of 154 patients shows a male predominance of 83% (*n* = 127), which is comparable in both groups as well, 70% (*n* = 30) in the ≤58 mm group and 84% (*n* = 97) in the >58 mm group, *p* = 0.511. There was no significant difference in racial distribution between the small and large LV groups, *p* = 0.302. Baseline comorbidities such as hypertension (55% vs. 53%, *p* = 0.823), diabetes (40% vs. 43%, *p* = 0.732), body mass index (BMI) (28.2 vs. 27.7 kg/m2, *p* = 0.650) and glomerular filtration rate (GFR) (48 vs. 51 mL/min/1.73m2), were also similar across groups.

**Table 1:** Baseline characteristics.

	Total Cohort (*n* = 154)	≤ 58 mm cohort (*n* = 38)	> 58 mm cohort (*n* = 116)	*p*-value
Age (Median, IQR)	58 (51–65)	60 (53–66)	57 (49–65)	0.196
Race (n, %)				
White	83 (54%)	24 (63%)	59 (51%)	0.302
African American	65 (42%)	12 (32%)	53 (46%)	
Other	6 (4%)	2 (5%)	4 (3%)	
Gender (n, %)				
Male	127 (83%)	30 (79%)	97 (84%)	0.511
Female	27 (18%)	8 (21%)	19 (16%)	
Etiology (n, %)				
Non-ischemic cardiomyopathy	108 (71%)	24 (63%)	84 (74%)	0.215
Ischemic cardiomyopathy	44 (29%)	14 (37%)	30 (26%)	
Baseline comorbidities				
Hypertension (n, %)	72 (54%)	21 (55%)	51 (53%)	0.823
Diabetes (n, %)	56 (42%)	15 (40%)	41 (43%)	0.732
Body mass index (BMI) (kg/m2) (median, IQR)	27.7 (24.3–31.3)	28.2 (25.0–30.9)	27.7 (24.0–32.4)	0.650
Glomerular filtration rate (eGFR) (mL/min/1.73m2) (median, IQR)	50.0 (39.5–73.0)	48.0 (37.5–65.5)	51.0 (40.0–75.0)	0.357
Baseline Ejection Fraction (%) (median, IQR)	18 (15–23)	24 (19–31)	16 (14–20)	**<0**.**001**
Left ventricular internal dimension in diastole (LVIDd) (mm) (median, IQR)	66 (59–74)	51 (47–55)	70 (65–77)	**<0**.**001**
Duration of Impella 5.5 support (days) (median, IQR)	24 (15–41)	24 (11–47)	24 (15–41)	0.832

BMI, body mass index; eGFR, estimated Glomerular filtration rate; LVIDd, Left ventricular internal dimension in diastole; IQR, interquartile range.

Bold values - statistically significant.

The median LVIDd in the ≤58 mm group was 51 mm (47–55) and in the >58 mm cohort was 70 mm (75–77). Baseline ejection fraction was significantly higher in the ≤58 mm cohort compared to the >58 mm cohort [25% (19–31) vs. 16% (14–20)], *p* < 0.001. Non-ischemic cardiomyopathy was the major etiology in both groups, 24 (63%) vs. 84 (74%), *p* = 0.215. Impella 5.5 device support duration is comparable across groups, 24 days (11–47) in the ≤58 mm group vs. 24 days (15–41) in the >58 mm group, *p* = 0.832.

### 3.2 Hemodynamics and laboratory results pre- and post-Impella 5.5 implantation

Both groups demonstrated similar trends and/or significant improvements in key hemodynamic variables at 72 h post-implantation (Table 2). In the ≤58 mm cohort, notable reductions were observed in pulmonary artery (PA) systolic pressure (54 to 36 mmHg, *p* < 0.001), PA diastolic pressure (29 to 15 mmHg, *p* < 0.001), mean PA pressure (37 to 24 mmHg, *p* < 0.001), and pulmonary capillary wedge pressure (PCWP) (27 to 19 mmHg, *p* = 0.004). Right atrial (RA) pressure also trended lower (12 to 10 mmHg, *p* = 0.073). Fick Cardiac output (CO) and Fick Cardiac index (CI) significantly increased post-Impella (4.00 to 5.85 L/min and 1.99 to 2.91 L/min/m2, both *p* < 0.001) accompanied by a modest rise in systemic venous oxygen saturation (SvO2), 60.2 to 63.9%, *p* = 0.032.

**Table 2:** Hemodynamics and laboratory results.

	≤ 58 mm cohort (*n* = 38) (median, IQR)			> 58 mm cohort (*n* = 116) (median, IQR)		
	Pre-Impella	Post-Impella (72 h)	*p*-value	Pre-Impella	Post-Impella (72 h)	*p*-value
Heart Rate (bpm)	85 (67–103)	84 (71–94)	0.485	88 (78–102)	92 (82–105)	**0**.**017**
Systolic BP (mmHg)	114 (99–124)	115 (98–127)	0.456	102 (97–111)	103 (95–114)	0.967
Diastolic BP (mmHg)	75 (67–88)	75 (69–81)	0.367	72 (66–78)	75 (66–81)	0.422
Mean Arterial Pressure (MAP) (mmHg)	90 (79–99)	87 (79–96)	0.600	83 (77–90)	85 (78–89)	0.459
Right Atrial (RA) pressure (mmHg)	12 (8–18)	10 (5–16)	0.073	10 (6–15)	7 (4–11)	**<0**.**001**
PA systolic (mmHg)	54 (38–61)	36 (25–46)	**<0**.**001**	53 (45–63)	48 (41–55)	**<0**.**001**
PA diastolic (mmHg)	29 (20–34)	15 (12–23)	**<0**.**001**	29 (23–33)	23 (19–26)	**<0**.**001**
PA mean (mmHg)	37 (27–42)	24 (19–31)	**<0**.**001**	37 (30–43)	31 (26–35)	**<0**.**001**
Pulmonary capillary wedge pressure (PCWP) (mmHg)	27 (19–31)	19 (14–23)	**0**.**004**	28 (21–31)	21 (17–26)	**0**.**006**
Fick Cardiac Output (L/min)	4.00 (3.39–4.84)	5.85 (4.90–7.28)	**<0**.**001**	3.60 (3.07–4.50)	5.70 (4.80–7.10)	**<0**.**001**
Fick Cardiac Index (L/min/m2)	1.99 (1.55–2.34)	2.91 (2.43–3.76)	**<0**.**001**	1.78 (1.50–2.20)	2.79 (2.33–3.25)	**<0**.**001**
Mixed venous oxygen saturation (SvO2) (%)	60.2 (52.2–67.0)	63.9 (58.8–69.4)	**0**.**032**	55.2 (47.3–63.2)	64.1 (57.1–70.7)	**<0**.**001**
Hematocrit (%)	32.2 (28.3–38.4)	28.7 (26.4–35)	**<0**.**001**	33.5 (28.6–38.8)	30.5 (26.8–35.1)	**<0**.**001**
Lactate Dehydrogenase (U/L)	-	269 (209–383)	-	-	276 (243–364)	-
Glomerular filtration rate (eGFR) (mL/min/1.73m2)	46 (30–59)	60 (43–90)	**<0**.**001**	50 (35–68)	66 (45–90)	**<0**.**001**

BP, blood pressure; eGFR, estimated glomerular filtration rate; MAP, mean arterial pressure; PA, pulmonary artery; PCWP, Pulmonary capillary wedge pressure; IQR, interquartile range.

Bold values - statistically significant.

Similar trends were observed in the >58 mm cohort, including significant reductions in RA pressure (10 to 7 mmHg, *p* = 0.001), mean PA pressures (37 to 31 mmHg *p* = 0.001), and PCWP (28 to 21 mmHg, *p* = 0.006). Fick CO improved from 3.60 to 5.70 L/min (*p* = 0.001), Fick CI improved from 1.78 to 2.79 L/min/m2 (*p* < 0.001) and SvO2 increased from 55.2 to 64.1% (*p* = 0.001).

GFR improved significantly in both cohorts post-Impella 5.5 implantation, 46 (30–59) to 60 (43–90) mL/min/1.73m2 in the ≤58 mm group and 50 (35–68) to 66 (45–90) mL/min/1.73m2 in the >58 mm group, both *p* < 0.001. A post-Impella 5.5 implant decrease in hematocrit was observed in both groups, 32.2 to 28.7% in the ≤58 mm group and from 33.5 to 30.5% in the >58 mm group (both *p* < 0.001). Lactate dehydrogenase (LDH) values were similar between groups post-implant, with medians of 269 U/L and 276 U/L, respectively.

### 3.3 Complications and outcomes

The majority of patients in both groups underwent heart transplantation, 30 (79%) in the ≤58 mm and 105 (90%) in the >58 mm cohort, *p* = 0.088 (Table 3). Durable LVAD implantation rates were also comparatively insignificant, 1(2%) in the ≤58 mm group and 7(6%) in the >58 mm group (*p* = 0.159). Interestingly, in our study population, native myocardial recovery was noted only in the ≤58 mm group, 4 (11%), *p* = 0.003. 30-day mortality was 6% in the ≤58 mm group and 3% in the >58 mm group, *p* = 0.078. 90-day mortality was 2% in both groups, *p* = 0.105.

**Table 3:** Outcomes of Impella 5.5 implantation.

	Total cohort (*n* = 154) (n, %)	≤ 58 mm cohort (*n* = 38) (n, %)	> 58 mm cohort (*n* = 116) (n, %)	*p*-value
Transplanted	134 (87.0%)	30 (79%)	104 (90%)	0.088
LVAD implantation	8 (5.2%)	1 (2%)	7 (6%)	0.159
Native Recovery	4 (2.6%)	4 (11%)	0 (0%)	**0**.**003**
30-day mortality	5 (3.2%)	2 (6%)	3 (3%)	0.078
90-day mortality	8 (5.2%)	3 (8%)	5 (4%)	0.105

LVAD, left ventricular assist device.

Bold values - statistically significant.

In the ≤58 mm group, three patients (8%) died after Impella 5.5 implantation: one succumbed to refractory non-convulsive status epilepticus that remained unresolved despite aggressive treatment, while two patients experienced severe multiorgan dysfunction and hypoperfusion, precluding further escalation of care. In terms of safety profile, our cohort had a 90-day event-free survival rate of 92%, with 35 out of 38 patients remaining event-free. There were no instances of major vascular compromise, major bleeding events (requiring >4 units pRBCs), stroke, device malposition, suction events or escalation to Bi-ventricular support before terminal therapy. Furthermore, none of the hypothesized complications for small LV patients such as LV wall injury, and mitral valvular apparatus injury were noted in our cohort. Clinically relevant hemolysis was seen in one patient who had multiple comorbidities including atrial fibrillation, chronic kidney disease stage 3b, low grade bladder malignancy and diverticulosis. His LDH increased to as high as 400 U/L, hematocrit as low as 19% and hemoglobin as low as 6 g/dL and he also suffered diverticular bleed that required intermitted pRBCs infusion.

### 3.4 Exploratory subgroup analysis using a stringent ≤52 mm cutoff

#### 3.4.1 Baseline characteristics

The median age was similar between groups, 60 (55–63) years in the ≤52 mm group vs. 57 (49–65) years in the >52 mm group, *p* = 0.196 (Table 4). Our study cohort of 154 patients shows a male predominance of 83% (*n* = 127), which is comparable in both groups as well, 81.0% (*n* = 17) in the ≤52 mm group and 82.7% (*n* = 110) in the >52 mm group, *p* = 0.511. There was no significant difference in racial distribution between the small and large LV groups, *p* = 0.302. Baseline comorbidities such as hypertension (47.6% vs. 54.9%, *p* = 0.823), diabetes (38.1% vs. 42.5%, *p* = 0.732), body mass index (BMI) (27.0 vs. 28.1 kg/m^2^, *p* = 0.650) and glomerular filtration rate (eGFR) (47.5 vs. 50.0 mL/min/1.73m^2^, *p* = 0.357), were also similar across groups.

**Table 4:** Baseline characteristics.

	Total Cohort (*n* = 154)	≤52 mm cohort (*n* = 21)	>52 mm cohort (*n* = 133)	*p*-value
Age (Median, IQR)	58 (51–65)	60 (55–63)	57 (49–65)	0.196
Race (n, %)				0.302
White	83 (54%)	13 (61.9%)	70 (52.6%)	
African American	65 (42%)	6 (28.6%)	59 (44.4%)	
Other	6 (4%)	2 (9.5%)	4 (3.0%)	
Gender (n, %)				0.511
Male	127 (83%)	17 (81.0%)	110 (82.7%)	
Female	27 (18%)	4 (19.0%)	23 (17.3%)	
Etiology (n, %)				0.215
Non-ischemic cardiomyopathy	108 (71%)	14 (66.7%)	94 (71.8%)	
Ischemic cardiomyopathy	44 (29%)	7 (33.3%)	37 (28.2%)	
Baseline comorbidities				
Hypertension (n, %)	72 (54%)	10 (47.6%)	62 (54.9%)	0.823
Diabetes (n, %)	56 (42%)	8 (38.1%)	48 (42.5%)	0.732
Body mass index (BMI) (kg/m^2^) (median, IQR)	27.7 (24.3–31.3)	27.0 (25.0–29.8)	28.1 (24.2–31.5)	0.650
Glomerular filtration rate (eGFR) (mL/min/1.73m^2^) (median, IQR)	50.0 (39.5–73.0)	47.5 (36.0–60.5)	50.0 (40.0–73.0)	0.357
Baseline Ejection Fraction (%) (median, IQR)	18 (15–23)	27 (18.3–36.0)	17 (15–20)	**<0**.**001**
Left ventricular internal dimension in diastole (LVIDd) (mm) (median, IQR)	66 (59–74)	49 (45–50)	69 (62–75)	**<0**.**001**
Duration of Impella 5.5 support (days) (median, IQR)	24 (15–41)	23 (11–37)	24 (15–41)	0.832

BMI, body mass index; eGFR, estimated Glomerular filtration rate; LVIDd, Left ventricular internal dimension in diastole; IQR, interquartile range.

Bold values - statistically significant.

The median LVIDd in the ≤52 mm group was 49 mm (45–50) and in the >52 mm cohort was 69 mm (62–75), *p* < 0.001. Baseline ejection fraction was significantly higher in the ≤52 mm cohort compared to the >52 mm cohort [27% (18.3–36.0) vs. 17% (15–20)], *p* < 0.001. Non-ischemic cardiomyopathy was the major etiology in both groups, 14 (66.7%) vs. 94 (71.8%), *p* = 0.215. Impella 5.5 device support duration is comparable across groups, 23 days (11–37) in the ≤52 mm group vs. 24 days (15–41) in the >52 mm group, *p* = 0.832.

#### 3.4.2 Hemodynamics and laboratory results pre- and post-Impella 5.5 implantation

Both groups demonstrated similar trends and/or significant improvements in key hemodynamic variables at 72 h post-implantation (Table 5). In the ≤52 mm cohort, notable reductions were observed in right atrial (RA) pressure (17 to 10 mmHg, *p* = 0.041), pulmonary artery (PA) systolic pressure (47 to 39 mmHg, *p* = 0.011), PA diastolic pressure (29 to 16 mmHg, *p* = 0.002), and mean PA pressure (36 to 26 mmHg, *p* = 0.008). Pulmonary capillary wedge pressure (PCWP) also trended lower (25 to 21 mmHg, *p* = 0.150). Fick Cardiac output (CO) and Fick Cardiac index (CI) significantly increased post-Impella (4.15 to 5.40 L/min, *p* = 0.026 and 1.98 to 2.78 L/min/m^2^, *p* = 0.013, respectively), while systemic venous oxygen saturation ($SvO_{2}$) remained relatively stable (63.9% to 63.2%, *p* = 0.279).

**Table 5:** Hemodynamics and laboratory results.

	≤52 mm cohort (*n* = 21)			>52 mm cohort (*n* = 133)		
	Pre-Impella	Post-Impella (72 h)	*p*-value	Pre-Impella	Post-Impella (72 h)	*p*-value
Heart Rate (bpm)	79 (66–98)	88 (72–99)	0.124	88 (77–102)	91 (80–102)	**0**.**050**
Systolic BP (mmHg)	119 (104–130)	111 (99–125)	0.132	102 (97–114)	105 (96–117)	0.893
Diastolic BP (mmHg)	80 (70–88)	74 (67–80)	0.057	72 (67–79)	75 (69–81)	0.381
Mean Arterial Pressure (MAP) (mmHg)	94 (83–99)	87 (83–96)	0.221	83 (77–90)	86 (79–92)	0.433
Right Atrial (RA) pressure (mmHg)	17 (8–22)	10 (9–13)	**0**.**041**	10 (6–15)	7 (4–11)	**<0**.**001**
PA systolic (mmHg)	47 (37–58)	39 (22–42)	**0**.**011**	54 (45–63)	47 (39–53)	**<0**.**001**
PA diastolic (mmHg)	29 (19–32)	16 (14–23)	**0**.**002**	29 (23–33)	22 (18–26)	**<0**.**001**
PA mean (mmHg)	36 (25–39)	26 (18–31)	**0**.**008**	37 (30–43)	30 (26–35)	**<0**.**001**
Pulmonary capillary wedge pressure (PCWP) (mmHg)	25 (16–31)	21 (14–23)	0.150	27 (22–31)	21 (17–25)	**<0**.**001**
Fick Cardiac Output (L/min)	4.15 (3.83–5.21)	5.40 (4.90–6.90)	**0**.**026**	3.66 (3.04–4.50)	5.70 (4.81–7.20)	**<0**.**001**
Fick Cardiac Index (L/min/m2)	1.98 (1.65–2.45)	2.78 (2.21–3.33)	**0**.**013**	1.78 (1.50–2.20)	2.81 (2.38–3.38)	**<0**.**001**
Mixed venous oxygen saturation (SvO2) (%)	63.9 (53.6–67.7)	63.2 (58.8–69.4)	0.279	55.5 (47.5–63.0)	64.6 (57.1–70.4)	**<0**.**001**
Hematocrit (%)	30.1 (28.3–37.1)	27.7 (24.5–34.3)	**0**.**004**	33.5 (28.6–38.9)	30.4 (26.8–35.1)	**<0**.**001**
Lactate Dehydrogenase (U/L)	-	260 (207–405)	-	-	276 (243–361)	-
Glomerular filtration rate (eGFR) (mL/min/1.73m2)	45 (23–56)	57 (49–90)	**0**.**003**	49 (33–68)	66 (44–90)	**<0**.**001**

BP, blood pressure; eGFR, estimated glomerular filtration rate; MAP, mean arterial pressure; PA, pulmonary artery; PCWP, Pulmonary capillary wedge pressure; IQR, interquartile range.

Bold values - statistically significant.

Similar trends were observed in the >52 mm cohort, including significant reductions in RA pressure (10 to 7 mmHg, *p* < 0.001), mean PA pressures (37 to 30 mmHg, *p* < 0.001), and PCWP (27 to 21 mmHg, *p* < 0.001). Fick CO improved from 3.66 to 5.70 L/min (*p* < 0.001), Fick CI improved from 1.78 to 2.81 L/min/m^2^ (*p* < 0.001), and $SvO_{2}$ increased from 55.5% to 64.6% (*p* < 0.001).

GFR improved significantly in both cohorts post-Impella 5.5 implantation, 45 (23–56) to 57 (49–90) mL/min/1.73m^2^ in the ≤52 mm group (*p* = 0.003) and 49 (33–68) to 66 (44–90) mL/min/1.73m^2^ in the >52 mm group (*p* < 0.001). A post-Impella 5.5 implant decrease in hematocrit was observed in both groups, 30.1% to 27.7% in the ≤52 mm group (*p* = 0.004) and from 33.5% to 30.4% in the >52 mm group (*p* < 0.001). Lactate dehydrogenase (LDH) values were similar between groups post-implant, with medians of 260 U/L and 276 U/L, respectively.

#### 3.4.3 Complications and outcomes

The majority of patients in both groups underwent heart transplantation, though the rate was significantly lower in the small LV group, 15 (71.4%) in the ≤52 mm cohort compared to 119 (89.5%) in the >52 mm cohort, *p* < 0.001 (Table 6). Durable LVAD implantation occurred only in the larger LV group, with 0 (0.0%) in the ≤52 mm group and 8 (6.0%) in the >52 mm group. Notably, all cases of native myocardial recovery in the entire study cohort occurred within the ≤52 mm group, representing 4 (19.0%) patients, compared to 0 (0.0%) in the >52 mm group. There were no statistically significant differences in short-term mortality between the groups; 30-day mortality was 4.8% (*n* = 1) in the ≤52 mm group and 3.0% (*n* = 4) in the >52 mm group (*p* = 0.673), while 90-day mortality was 9.5% (*n* = 2) in the ≤52 mm group and 4.5% (*n* = 6) in the >52 mm group (*p* = 0.336).

**Table 6:** Outcomes of Impella 5.5 implantation.

	Total cohort (*n* = 154) (n, %)	≤52 mm cohort (*n* = 21) (n, %)	>52 mm cohort (*n* = 133) (n, %)	*p*-value
Transplanted	134 (87.0%)	15 (71.4%)	119 (89.5%)	<0.001
LVAD implantation	8 (5.2%)	0 (0.0%)	8 (6.0%)	
Native Recovery	4 (2.6%)	4 (19.0%)	0 (0.0%)	
30-day mortality	5 (3.2%)	1 (4.8%)	4 (3.0%)	0.673
90-day mortality	8 (5.2%)	2 (9.5%)	6 (4.5%)	0.336

LVAD, left ventricular assist device.

The smallest LVIDd in this our study population was 40 mm in a 61-year-old male with multiple comorbidities including multiple myeloma complicated by AL amyloid cardiomyopathy in remission, heart failure with reduced ejection fraction 37% and profoundly elevated PA pressures 68/33 mmHg, treated with multiple MCS devices prior including intra-aortic balloon pump and Tandem heart. This patient underwent a successful heart transplant following 24 days of Impella 5.5 support without complications.

## 4 Discussion

With the growing use of Impella 5.5, it is crucial to closely assess risk factors for poor outcomes and candidacy, including anatomical factors such as baseline LV size.

Our data highlights a gap in the current knowledge of managing critically ill patients with cardiogenic shock requiring temporary mechanical circulatory support as a bridge to decision. We provide much-needed insight within our population of patients beyond just left ventricular dimension and build upon our previously published data showing the improvement and optimization in a multi-factorial way: renal recovery, pulmonary hemodynamic optimization and exceptional short- and long-term outcomes with a variety of terminal therapies, including extended Impella 5.5 support in our cohort (4–7).

While data on this specific population remains limited, a case report has demonstrated safe use of Impella 5.5 in a 50-year-old female patient with left ventricular non-compaction syndrome, who was deemed ineligible for LVAD placement due to small LV size (3). This patient tolerated Impella 5.5 support for 120 days without complications before undergoing a successful heart transplant. These findings add to the growing evidence supporting the device's role in patients with HF; however, no prior studies have systematically evaluated safety, efficacy, complications, and outcomes associated with Impella 5.5 in patients with a small LV cavity (using either the traditional ≤58 mm cutoff or the more stringent clinical cutoff of ≤52 mm). Our study represents the first comprehensive analysis of these subsets of heart failure (HF) patients.

Furthermore, we are also able to confidently state that the Impella 5.5's safety in patients with cardiogenic shock managed by a skilled multidisciplinary team has been demonstrated at our institution, regardless of support duration, with or without a small left ventricular cavity. Our discussion below focuses on the small LV cavity physiology and patient outcomes, building upon the limited amount of available data that has been peer-reviewed and published.

### 4.1 Outcomes and therapeutic trajectories

Our data demonstrates excellent, comparable survival outcomes of Impella 5.5 implantation across both groups, irrespective of the pre-Impella implantation LV size. The small LV cohort (LVIDd ≤58 mm) had a robust 90-day survival rate of 92%, supporting the safety profile and feasibility of the use of Impella 5.5 in patients with small LV, while also showing great outcomes in terms of the success rate as a bridge to transplant (79%), and native recovery (14%). Patients with LV size >58 mm showed similar outcomes: 104 (90%) transplanted (*p* = 0.088), 7 (6%) durable LVAD implantation (*p* = 0.159) and 90-day survival rate of 96% (*p* = 0.105).

Importantly, this therapeutic success is maintained even when evaluating the more stringent, informally accepted clinical cutoff of ≤52 mm. Within this extremely small LV cohort, 90-day survival remained excellent at 90.5% (*p* = 0.336), proving that spatial constraints do not compromise overall survival. While the rate of heart transplantation was lower in the ≤52 mm group compared to the larger LV group (71% vs. 90%, *p* < 0.001), this difference was not driven by mortality. Instead, it is explained by the fact that all cases of native myocardial recovery in the entire study cohort occurred within the ≤52 mm group (19.0% vs. 0.0%). Furthermore, none of the patients in the ≤52 mm group required escalation to durable LVAD support (0% vs. 6%).

This suggests that patients with smaller, non-dilated ventricles (median LVIDd of 49 mm and a significantly higher baseline ejection fraction of 27%) preserve a highly viable myocardial function capable of reverse remodeling and functional recovery under the protective, unloading conditions of the Impella 5.5. Conversely, no patients in the ≤52 mm group received a durable LVAD (0% vs. 6.0% in the >52 mm group) suggesting that patients with larger, severely dilated ventricles represent a patient population with end-stage, irreversible remodeling where recovery is no longer a viable pathway, necessitating transition to transplant or durable LVAD.

Both cohorts demonstrate significant improvements in hemodynamic parameters post-implantation, further supporting the device's efficacy in stabilizing patients with cardiogenic shock across a wide range of LV sizes.

### 4.2 Preload and afterload optimization

In our original ≤58 mm cohort, RA pressure decreased by 2 mmHg (12 to 10 mmHg) in the ≤58 mm cohort without reaching statistical significance, whereas the larger ventricle group had a more consistent and significant 3 mmHg reduction (10 to 7 mmHg), suggesting more predictable venous unloading in patients with larger ventricles. However, when isolating the narrower ≤52 mm cohort, we observed highly consistent, statistically significant systemic and pulmonary venous unloading. Specifically, in the ≤52 mm group, RA pressure decreased significantly from 17 to 10 mmHg (*p* = 0.041), matching the significant reductions seen in the larger ventricle group (10 to 7 mmHg, *p* < 0.001).

The hemodynamic responses observed at 72 h post-implantation in the ≤ 52 mm cohort provide strong physiological proof of safe, effective left ventricular unloading without inducing suction or collapse. In the ≤ 52 mm cohort, we observed highly statistically significant reductions across all primary right- and left-sided pressure indices. Pulmonary capillary wedge pressure (PCWP) decreased in both groups, [trending lower in the ≤52 mm group (25 to 21 mmHg, *p* = 0.150) and dropping significantly in the >52 mm cohort (27 to 21 mmHg, *p* < 0.001). This may support the idea that smaller ventricles experience greater preload sensitivity because of higher baseline filling pressures. Clinically, this may suggest that smaller ventricles may show larger fluctuations in filling pressures in response to volume changes, requiring more precise preload management compared to those with a dilated LV cavity. Crucially, this substantial unloading occurred alongside a highly significant improvement in systemic forward flow within the ≤52 mm cohort: Fick CO improved from 4.15 to 5.40 L/min (*p* = 0.026) and Fick CI improved from 1.98 to 2.78 L/min/m^2^ (*p* = 0.013). This simultaneous reduction in right-sided filling pressures and marked increase in systemic output suggests that active unloading of the LV benefits the right ventricle (RV). By mitigating downstream pulmonary congestion and reducing RV afterload, the Impella 5.5 successfully breaks the cycle of biventricular failure, even within highly confined intraventricular spaces.

Although afterload reduction is not explicitly presented here, the thought of recoupling should be considered in future analyses to better understand the relationship between pre-Impella LV sizes and hemodynamic response, improving our understanding of patient-specific preload and afterload management.

### 4.3 Renal function trends

The baseline eGFR in the entire cohort was 50 mL/min/1.73m2 (39–73) corresponding primarily to chronic kidney disease (CKD) stage 3a−3b. There was no significant difference at baseline between the subgroups in either analysis (≤58 mm vs. > 58 mm: 48 vs. 51 mL/min/1.73m^2^, *p* = 0.357; ≤52 mm vs. > 52 mm: 47.5 vs. 50 mL/min/1.73m^2^, *p* = 0.357. However, at only 72 h post-Impella, both groups demonstrated significant improvement in eGFR, with the ≤58 mm cohort increasing from 46 (30–59) to 60 (43–90) mL/min/1.73m2, *p* < 0.001, and the >58 mm cohort increasing from 50 (35–68) to 66 (45–90) mL/min/1.73m2, *p* < 0.001. This renal recovery was replicated with equal clinical efficacy in the $\le$52 mm subgroup, where median eGFR increased significantly from 45 (23–56) to 57 (49–90) mL/min/1.73m^2^ (*p* = 0.003), while the >52 mm cohort improved from 49 (33–68) to 66 (44–90) mL/min/1.73m^2^ (*p* < 0.001). Both groups effectively improved from CKD stage 3b to 3a/2, irrespective of pre-Impella LV cavity size.

A primary clinical concern of placing a microaxial flow pump within a restricted LV cavity is the potential for inflow obstruction or suction, which would severely compromise forward cardiac output and worsen end-organ perfusion. These findings suggest this concern may be insignificant, taking into consideration robust multidisciplinary and surgical caliber of the center. This further supports our group's findings that Impella 5.5 leads to meaningful clinical recovery in renal function, perhaps, even mitigating the need for renal replacement therapies, which we have previously published.

### 4.4 Physiology of adverse events

Smaller LV volumes are associated with unfavorable intraventricular hemodynamics, which may cause platelet shearing, leading to platelet activation and aggregation. The resulting increased thrombogenicity may lead to associated complications such as cerebrovascular events and arrhythmias (8). Furthermore, an intricate balance between LV preload, LV size and LVAD speed is crucial for maintaining adequate blood flow in continuous-flow devices such as the Impella 5.5 and durable LVADs (8). Evidence suggests that LV size is the strongest factor contributing to an increased risk of thrombosis in patients with LVADs (8).

However, there is limited literature on intraventricular blood flow dynamics in patients with continuous flow devices. A 2022 study consisting of 313 patients who underwent durable LVAD were found to have worse survival if LVEDD size was <59 mm. Smaller LVEDD patients had lower survival compared to larger LVEDD patients (71% vs. 85% at 1 year and 58% vs. 80% at 2 years, *p* = 0.003) (2).

Comparatively, our study, consisting of comparable baseline demographics, comorbidities and laboratory data between the two groups, demonstrates the safety of Impella 5.5 in patients with LVIDd as small as 40 mm. Despite an average LVIDd of only 49 mm in our smaller subgroup, we did not encounter any known complications of Impella 5.5 use (major vascular compromise, bleeding, stroke, device malposition, suction events or escalation to Bi-ventricular support before terminal therapy), nor the theoretical complications that may occur in patients with small LV (LV wall injury, and mitral valvular apparatus injury).

This safety profile is supported by our post-implant laboratory markers. While a post-Impella 5.5 implant decrease in hematocrit was observed in both groups (30.1% to 27.7% in the ≤52 mm group, *p* = 0.004; 33.5% to 30.4% in the >52 mm group, *p* < 0.001), median LDH values post-implant were similar and remained low between groups (260 U/L in the ≤52 mm group vs. 276 U/L in the >52 mm group), indicating no clinically significant device-induced hemolysis or pathological platelet shearing.

### 4.5 Safety considerations and monitoring with Impella 5.5 use

While outcomes in our cohort were favorable, careful peri-procedural investigations are required to mitigate potential complications in this cohort. In a study from the Food and Drug Administration (FDA) Manufacturer and User Facility Device Experience (MAUDE) database which evaluates all Impella devices, 50 events of ventricular perforations were reported over the period of 2009–2021. Of these 11(22%) occurred in patients with Impella 5.5. These were caused primarily during the process of device repositioning (24%), followed by cardiac surgery (14%) and finally, guidewire related wall injury (14%). The underlying cause was hypothesized to be ischemia-induced myocardial necrosis (24%), which weakens the LV wall and predisposes to injuries and perforation (9). Hence, thorough peri-procedural planning is essential to reduce the risk of complications. Imaging guidance should be utilized to ensure accurate positioning to avoid wall injury in patients with small LV sizes. Regular monitoring of hematocrit, lactate dehydrogenase, and platelet levels is crucial to detect hemolysis and signs of platelet activation, helping minimize the risk of thrombosis and associated vascular complications.

## 5 Limitations

Our study is limited by the sample size and single-center design. Multicenter studies with a larger sample size may be required to definitively assess the use of Impella in patients with small LV and potentially establish enhanced device management protocols and anticoagulation strategies. Data presented here did trend toward significant, with a larger cohort of small LV patients likely to make it more robust. In the era of using temporary mechanical circulatory support, our center's experience provides insight and hypothesis-generating findings, as well as protocols for larger trials to be designed and implemented.

## 6 Conclusions

This study represents the largest sample of Impella 5.5 devices placed in small LV cavities, demonstrating robust survival, safety and feasibility with support beyond 14 days in this high-risk population with small LV cavities. Crucially, our subgroup analysis reveals that pushing to a stringent ≤52 mm threshold remains highly safe, while uniquely unlocking native myocardial recovery (19% vs. 0% in larger cavities). These findings challenge conventional clinical hesitation, proving that temporary microaxial support is highly effective even in extremely anatomically constrained ventricles.

## Data Availability

The raw data supporting the conclusions of this article will be made available by the authors, without undue reservation.
